# Sentinel Surveillance: A Reliable Way To Track Antibiotic Resistance in Communities?

**DOI:** 10.3201/eid0805.010268

**Published:** 2002-05

**Authors:** Stephanie J. Schrag, Elizabeth R. Zell, Anne Schuchat, Cynthia G. Whitney

**Affiliations:** * Centers for Disease Control and Prevention, Atlanta, Georgia, USA

**Keywords:** *Streptococcus pneumoniae*, antimicrobial resistance, surveillance

## Abstract

We used population-based data to evaluate how often groups of randomly selected clinical laboratories accurately estimated the prevalence of resistant pneumococci and captured trends in resistance over time. Surveillance for invasive pneumococcal disease was conducted in eight states from 1996 to 1998. Within each surveillance area, we evaluated the proportion of all groups of three, four, and five laboratories that estimated the prevalence of penicillin-nonsusceptible pneumococci (%PNSP) and the change in %PNSP over time. We assessed whether sentinel groups detected emerging fluoroquinolone resistance. Groups of five performed best. Sentinel groups accurately predicted %PNSP in five states; states where they performed poorly had high between-laboratory variation in %PNSP. Sentinel groups detected large changes in prevalence of nonsusceptibility over time but rarely detected emerging fluoroquinolone resistance. Characteristics of hospital-affiliated laboratories were not useful predictors of a laboratory’s %PNSP. Sentinel surveillance for resistant pneumococci can detect important trends over time but rarely detects newly emerging resistance profiles.

Antibiotic-resistant infections are an emerging problem in community as well as nosocomial settings. *Streptococcus pneumoniae* infections are a leading cause of community-acquired respiratory illness in young children, the elderly, and persons with chronic medical conditions. Pneumococcal infections range from otitis media and bacteremia to pneumonia and meningitis. Although penicillin has traditionally been an effective treatment for pneumococcal infections, in recent years the increasing prevalence of drug-resistant pneumococci threatens the effectiveness of antibiotic therapy (*1*,*2*).

Surveillance for resistant pneumococci is an essential component of public health efforts to prevent the spread of drug resistance. In addition to increasing awareness of the public and health-care providers about resistance, surveillance data can be used to target high-prevalence areas for judicious use of antibiotics, pneumococcal vaccination campaigns, or both; identify newly emerging strains and resistance profiles; and assess trends in resistance. At the national level, surveillance data can contribute to the development of clinical guidelines for managing pneumococcal disease (*3*,*4*). Local surveillance data can in some instances guide patient care (*4*).

The prevalence of drug-resistant pneumococci varies geographically. Because national trends may not reflect trends within specific regions, local and state-specific data can motivate prevention efforts (*5*). Although invasive disease due to drug-resistant pneumococci was added to the National Notifiable Diseases List in 1994, mandatory reporting remains low (53% of states and territories in 1999) (*6*), in part because collecting antimicrobial susceptibility data can be difficult. Active, population-based surveillance for resistant pneumococci based on laboratory-confirmed invasive disease may be considered the most accurate method of estimating rates of drug-resistant pneumococcal disease in a defined area. Such systems, however, are often costly and labor-intensive for state or local health departments to maintain.

Sentinel surveillance, a system that collects information on drug-resistant pneumococci from a limited sample of hospital, clinic, and/or private laboratories, has been suggested as a feasible alternative method of collecting regional data, and some states are adopting this approach (*7*). Although sentinel systems are useful for monitoring trends in a number of diseases (*8*–*10*) and a sentinel hospital surveillance system in the 1980s first detected increases in the prevalence of penicillin-resistant pneumococci in the United States (*11*), observations that the prevalence of resistant pneumococcal isolates can vary dramatically from laboratory to laboratory within a state or area (*12*) raise the question of whether sentinel laboratories can accurately reflect an area’s prevalence of pneumococcal resistance.

For pneumococcus, the most common approach to sentinel surveillance is to select a small number of clinical laboratories within an area and collect information on susceptibility of all invasive pneumococcal isolates at those facilities as a way of estimating the prevalence of resistance in the area as a whole. To evaluate the validity of this sentinel approach, we assessed how often small groups of laboratories in a given area accurately estimated the area’s proportion of resistant invasive pneumococcal isolates, using population-based surveillance as the standard. We also evaluated whether such sentinel groups of laboratories accurately tracked changes in the proportion of drug-resistant pneumococci over time, and whether they could detect newly emerging resistance profiles. Finally, we explored whether hospital characteristics could be used to guide selection of hospital laboratories for inclusion in sentinel systems, in order to increase the system’s representativeness and reliability.

## Methods

### Population-Based Data

Invasive pneumococcal surveillance was conducted from 1996 to 1998 as part of the Active Bacterial Core Surveillance/ Emerging Infections Program Network (ABCs) using previously described methods (*1*). Briefly, project personnel communicated at least twice each month with contacts in all participating microbiology laboratories serving acute-care hospitals in San Francisco County, California; Connecticut; eight counties in Georgia (Cobb, Clayton, De Kalb, Douglas, Fulton, Gwinnett, Newton, and Rockdale) with 12 additional Atlanta-area counties starting in 1997; six counties in Maryland (Anne Arundel, Baltimore, Baltimore City, Carroll, Harford, and Howard); seven counties in Minnesota (Anoka, Carver, Dakota, Hennepin, Ramsey, Scott, and Washington); seven counties in New York starting in 1997 (Genesee, Livingston, Monroe, Ontario, Orleans, Wayne, and Yates); three counties in Oregon (Clackamas, Multnomah, and Washington); and five counties in Tennessee (Davidson, Hamilton, Knox, Shelby, and Williamson).

A case was defined as the isolation of *Streptococcus pneumoniae* from a normally sterile site (e.g., blood or cerebrospinal fluid) from a resident of a surveillance area. Periodic audits were conducted in each area. Any cases newly identified by audits were included in the surveillance database.

All isolates were sent to one of two centralized laboratories for susceptibility testing by broth microdilution, with a panel of drugs that included (in 1998) penicillin, amoxicillin, cefotaxime, cefuroxime, meropenem, erythromycin, clindamycin, chloramphenicol, vancomycin, rifampin, levofloxacin, trovafloxacin, and quinupristin-dalfopristin (Synercid7). Nonsusceptibility (resistance and intermediate susceptibility) was determined according to criteria of the National Committee for Clinical Laboratory Standards (*13*).

### Ability of Sentinel Laboratory Groups To Estimate Proportion of Resistant Isolates

In each surveillance area for 1998, we generated all possible simple random samples of three, four, and five laboratories, excluding laboratories with <10 isolates. We limited our selection to up to five laboratories because a central objective of sentinel surveillance is to reduce required resources by reducing the number of facilities participating in the surveillance system. We refer to these simple random samples as sentinel groups of laboratories. We then calculated the percent of penicillin-nonsusceptible (MIC >0.1 µg/mL) pneumococci (%PNSP) among isolates in each of these sentinel groups and compared these percentages to the area’s actual %PNSP, as measured by ABCs. The %PNSP in sentinel groups was considered to be accurate if it was within 5 percentage points of the area’s actual %PNSP. We chose this interval because variation in the %PNSP within this range is unlikely to influence public health decisions (*12*).

We used a finite population correction based on the total number of isolates in each surveillance area to assess the number of randomly sampled isolates that would be needed to estimate an area’s actual %PNSP within 5 percentage points (*14*). We compared that number with the number of isolates in sentinel groups in each area.

### Ability of Sentinel Groups To Track Changes in Prevalence of Drug-Resistant Pneumococci over Time

In each surveillance area, we subtracted the %PNSP in each possible group of five laboratories in 1996 from that measured for the group of five laboratories in 1998. We included only laboratories with >10 isolates in each of the 2-year periods. We then measured how often the change in %PNSP in sentinel groups was within 5 percentage points of the area’s actual change in %PNSP during the same time periods, based on ABCs data. We performed a similar analysis using the percentage of erythromycin-nonsusceptible (MIC >0.5 µg/mL) isolates as the outcome measure.

### Ability of Sentinel Goups To Detect Emerging Fluoroquinolone Resistance

Using data from 1998, we measured the proportion of all possible groups of five sentinel laboratories within each surveillance area that captured any pneumococcal isolates with fluoroquinolone (levofloxacin or trovafloxacin) nonsusceptibility. We then compared that proportion with area-specific data on the presence of pneumococcal fluoroquinolone resistance from ABCs in 1998.

### Evaluation of Hospital Predictors of %PNSP

We merged ABCs data from 1997 and 1998 with purchased data on hospital characteristics collected by the American Hospital Association (AHA) as part of the AHA Annual Survey of Registered American Hospitals in 1997. We categorized each hospital that matched between the two datasets into the following PNSP classes: >5 percentage points above the surveillance area proportion PNSP (high PNSP), <5 percentage points above or below the surveillance area PNSP (average PNSP), or >5 percentage points below the surveillance area PNSP (low PNSP). We used logistic regression to perform univariate analyses. We compared hospital characteristics in the high group with those in the average group, separately comparing hospital characteristics in the low group with those in the average group. We categorized continuous variables according to their quartiles or medians based on their distributions. We limited our analysis to hospital characteristics that might plausibly influence a hospital’s %PNSP based on findings of previous studies (*15*,*16*).

## Results

### Population-Based Data

The %PNSP across surveillance areas in 1998 varied from 15 (California and New York) to 35 (Tennessee) (Table 1). The number of laboratories that isolated invasive pneumococci and the total number of invasive pneumococcal isolates also varied by surveillance area (Table 1). Consistent with previous observations (*12*), each surveillance area had striking variation across laboratories in the %PNSP in invasive pneumococcal isolates (Figure).

**Table 1:** Ability of sentinel groups of three, four, and five laboratories to estimate accurately %PNSP, 1998^a^

Area	Labs with >10 isolates (total labs)	Actual %PNSP	Total isolates	Percent of sentinel groups within 5 percentage points of actual %PNSP		
				3 labs (no. of groups; overall range in %PNSP)	4 labs (no. of groups; overall range in %PNSP)	5 labs (no. of groups; overall range in %PNSP)
CA	5 (9)	15	181	100 (10; 12-17)	100 (5; 13-16)	100 (1; NA)
CT	25 (32)	18	681	73 (2,300; 2-31)	81 (12,650;4-30)	87 (53,130;6-30)
GA	18 (34)	33	860	45 (816; 19-51)	52 (3,060; 20-49)	58 (8,568; 21-48)
MD	20 (26)	22	579	60 (1,140; 8-40)	68 (4,845; 9-38)	74 (15,504; 10-37)
MN	12 (24)	20	470	78 (220; 11-30)	88 (495; 12-29)	94 (792; 14-28)
NY	5 (19)	15	191	80 (10; 9-15)	100 (5; 10-14)	100 (1; NA)
OR	6 (13)	21	228	80 (20; 14-25)	93 (15; 14-23)	100 (6; 17-21)
TN	20 (30)	35	419	37 (1,140; 11-62)	40 (4,845; 13-59)	44 (15,504;14-57)

^a^In Active Bacterial Core surveillance areas. %PNSP, percent of penicillin-nonsusceptible invasive pneumococcal isolates.

**Figure:**
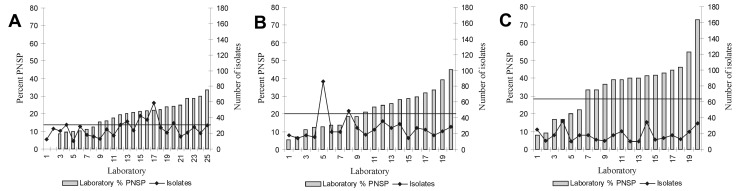
Between-laboratory variation in percent penicillin-nonsusceptible isolates (%PNSP) and number of invasive pneumococcal isolates in selected surveillance areas. A) Connecticut; B) Selected counties of Maryland; C) Selected counties of Tennessee. Solid line denotes the area’s actual %PNSP from active, population-based surveillance.

### Ability of Sentinel Laboratory Groups To Estimate %PNSP

In New York, California, and Oregon (areas with a relatively small number of laboratories with >10 invasive pneumococcal isolates), sentinel groups of three, four, or five laboratories all did well at estimating the area’s actual %PNSP (Table 1). In the remaining areas, increasing the number of laboratories included in sentinel groups from three to five increased the probability that the sentinel %PNSP approached the area’s actual %PNSP. However, in Georgia and Tennessee, the two areas with the highest actual %PNSP, sentinel groups of five laboratories still poorly estimated the area’s actual percentage (Table 1).

In surveillance areas where most sentinel groups had an adequate sample size to estimate %PNSP accurately (i.e., the number of isolates met the sample size requirement), sentinel groups performed well compared with population-based surveillance (Table 2). In contrast, in Georgia and Tennessee, where sentinel groups performed poorly, a smaller proportion of sentinel groups met the minimum sample size requirements. However, in some states that failed to meet sample size requirements (e.g., Connecticut), sentinel groups performed well.

**Table 2:** Number of isolates required to estimate accurately %PNSP in a given area and percentage of sentinel laboratory groups that met sample size requirements

Area	Actual %PNSP (target range)	No. of isolates needed to estimate %PNSP^a^	% of sentinel groups of 5 laboratories with > no. of required isolates
CA	15 (10-20)	94	100
CT	18 (13-23)	172	3
GA	33 (28-38)	243	40
MD	22 (17-27)	183	12
MN	20 (15-25)	163	70
NY	15 (10-20)	97	100
OR	21 (16-26)	120	100
TN	35 (30-40)	191	0

^a^ No. of isolates, n, required to estimate the area’s actual %PNSP (P) within 5 percentage points (d=0.05) with 95% confidence (Z=1.96) is: n= (Z^2^ P(1-P))/d^2^, where d is the range of accepted variation around the actual %PNSP, and Z is the Z-score range within which values must fall. Because the total no. of isolates per area, N, was small, we corrected this estimate for finite population size: n=n/[1+(n-1)/N]. There is no power associated with this estimate (14). %PNSP, percent of penicillin-nonsusceptible pneumocooccal isolates.

### Ability of Sentinel Groups To Detect Trends in Prevalence of Nonsusceptible Pneumococci

The actual change in %PNSP in 1998 compared with that in 1996 varied across areas, ranging from Georgia’s 2% decline to Maryland’s 7% increase (Table 3). Because sentinel groups of five were the most accurate at predicting an area’s actual %PNSP, we focused strictly on groups of five for this analysis. Laboratories participating in ABCs in 1998 were often not the same as those participating in 1996 because of hospital or laboratory mergers, closing or opening of microbiology facilities in the surveillance areas, and expansion of areas under surveillance. Consequently, only a subset of all possible sentinel groups in 1998 matched those in 1996.

**Table 3:** Ability of sentinel groups of five laboratories to estimate an area’s change in %PNSP and erythromycin-nonsusceptible pneumococci, 1996–1998

Outcome measure	Area^a^	Actual change in % NS pneumococci	% sentinel groups within 5 percentage points of the area’s actual change in % NS pneumococci	Percent of sentinel groups detecting an increase or decrease in the actual % NS pneumococci^c^
Penicillin NS	CA	+3	100 (1)	100
	CT	+1	67 (15,504)	
	GA	-2	76 (2,002)	
	MD	+7	70 (15,504)	93
	MN	+6	97 (252)	99
	TN	0	45 (462)	
Erythromycin NS	CA	-2	100 (1)	
	CT	+2	95 (15,504)	
	GA	+6	80 (2,002)	86
	MD	+6	97 (15,504)	99
	MN	+7	83 (252)	99.6
	TN	+2.5	51 (462)	--

^a^ NY joined ABCs in 1997; the only group of 5 laboratories in OR in 1996 did not match any of the groups in 1998. ^b^Groups that merged between the 2 years. ^c^We limited this analysis to areas with >3% change in either direction. %PNSP, percent penicillin-nonsusceptible pneumococci; NS, nonsusceptible.

Over two thirds of each area’s sentinel groups of five accurately estimated changes in %PNSP, except in Tennessee, where only 45% correctly estimated a <5 percentage point change (Table 3). In the three areas with large changes in %PNSP (>3 percentage points), >90% of sentinel groups in each area predicted the direction of the change (increases in each case).

Trends in the proportion of isolates that were erythromycin nonsusceptible also varied by area, and three areas showed large increases from 1996 to 1998 (Table 3). Similar to trends observed for penicillin nonsusceptibility, sentinel groups had a high probability of detecting these increases in erythromycin nonsusceptibility (Table 3).

### Ability of Sentinel Groups To Detect Emerging Fluoroquinolone Resistance

In 1998, seven isolates submitted to ABCs were nonsusceptible to levofloxacin; five of these were also nonsusceptible to trovafloxacin. The isolates came from seven different hospitals, located in five of the eight surveillance areas (California, Connecticut, Maryland, Minnesota, and Oregon). One of these hospitals, the only hospital from Oregon, had only five invasive pneumococcal isolates in 1998 and thus was excluded from our analysis of sentinel groups. Approximately 40% of sentinel groups of five laboratories in these areas (range 37% in Connecticut to 45% in Maryland) included a laboratory with a fluoroquinolone-nonsusceptible isolate, except in California, where there was only one possible sentinel group of five laboratories and this group included the fluoroquinolone-nonsusceptible isolate.

### Evaluation of Hospital Predictors of %PNSP

The merged dataset of ABCs and AHA hospitals contained 104 hospitals: 24 (23%) were in the high PNSP category, 52 (50%) were in the average PNSP category; and 28 (27%) were in the low PNSP category. Hospitals that admitted only children (four hospitals that matched between the two datasets) were significantly more likely to be in the high PNSP group than in the average group (all four hospitals fell in the high category; Fisher’s exact test, p=0.008). Larger hospitals (measured by adjusted inpatient days, total beds, or total beds set up and staffed) were more likely to fall in the average category, but this trend was not consistent for all indicators capturing hospital size (Table 4). Additional variables tested by univariate analysis were not predictive of falling in the high or low category (Table 4). When we performed similar analyses using the percent of erythromycin-nonsusceptible isolates or of isolates with resistance to more than one drug class as the primary outcome measure, no additional predictors were identified.

**Table 4:** Univariate analysis of characteristics of hospitals with a high or low %PNSP compared with hospitals with an average %PNSP^a^

High vs. average %PNSP					Low vs. average %PNSP				
Hospital characteristic	No.		Odds ratio	p value	No.			Odds ratio	p value
	High	Avg			Low		Avg		
Adjusted inpatient days^b^				0.02					0.06
0-66,452	11	7	Ref^c^		8		7	Ref	
66,453-104,771	5	11	0.29	0.09	10		11	0.80	0.73
104,772-146,879	6	17	0.23	0.03	3		17	0.15	0.02
>146,879	3	16	0.12	0.007	7		16	0.38	0.17
Total beds set up and staffed				0.04					0.25
0-173	11	8	Ref		7		8	Ref	
174-300	6	11	0.40	0.18	10		11	1.04	0.96
301-413	4	16	0.19	0.02	5		16	0.36	0.16
>414	4	16	0.19	0.02	6		16	0.43	0.23
Adult medical/surgical and ICU beds									
0-16	15	19	Ref		17		19	Ref	
>16	7	26	0.31	0.05	9		29	0.39	0.06
Pediatric medical/surgical and ICU beds									
0-10	13	20	Ref		16		20	Ref	
>11	9	25	0.55	0.26	10		25	0.50	0.17
Hospital with a pediatric ICU									
No	18	35	Ref		22		35	Ref	
Yes	4	10	0.78	0.70	4		10	0.64	0.49
Medicaid inpatient days				0.10					0.36
0-3,730	9	10	Ref		7		10	Ref	
3,731-8,797	7	10	0.78		9		10	1.3	0.71
8,798-19,477	7	15	0.52		4		15	0.38	0.20
>19,477	2	16	0.14		8		16	0.71	0.61
Medicare inpatient days				0.04					0.02
0-18,246	10	6	Ref		10	6		Ref	
18,247-29,026	5	12	0.25	0.06	9	12		0.45	0.24
29,027-45,471	5	18	0.17	0.01	3	18		0.10	0.005
>45,471	5	15	0.20	0.03	6	15		0.24	0.04
Metropolitan statistical area size									
<1 million population	5	10	Ref		5	10		Ref	
>l million population	20	41	0.98	0.97	23	41		1.12	0.84

^a^High %PNSP was defined as >5 percentage points above the surveillance area % of penicillin-nonsusceptible pneumococci (PNSP) ; low as >5 percentage points below the surveillance area %PNSP; average as <5 percentage points above or below the surveillance area %PNSP. ^b^Adjusted inpatient days were calculated as Inpatient Days + (Inpatient Days * [Outpatient Revenue/Inpatient Revenue]). ^c^Ref=Referent group. ICU, intensive-care unit.

In areas where sentinel surveillance did not accurately estimate the %PSNP (Georgia and Tennessee), can hospital predictors be used to improve performance? When we limited sentinel groups of five to the laboratories with the largest number of isolates, the range in %PNSP narrowed, but accuracy was not guaranteed (range in Georgia 29%-34%; range in Tennessee 36%-44%). Additionally, consistent with the analysis above, hand-picking sentinel hospitals to include those with a high proportion of pediatric isolates was likely to overestimate the actual %PNSP; in Georgia the children’s hospital had a %PNSP of 61%, whereas the area’s true %PNSP was 33% (Table 1).

## Discussion

As the incidence of drug-resistant pneumococcal disease continues to increase, the need for local and state-specific data on the emergence of drug-resistant invasive pneumococcal strains also grows. Although active, population-based surveillance provides highly accurate data for tracking pneumococcal resistance trends, few states can afford to implement such labor-intensive and costly systems. Moreover, states may have a variety of objectives for their surveillance systems, ranging from increasing awareness of resistance in local communities and promoting appropriate antibiotic use activities to estimating directly the drug-resistant isolates and trends in drug resistance; some of these objectives require more accurate surveillance systems than others.

Our evaluation of the performance of sentinel laboratory groups suggests that sentinel surveillance is a viable alternative to population-based surveillance in situations where a high degree of accuracy is not required. In some cases, sentinel surveillance may also be useful when accurate estimates of %PNSP trends are a primary objective. Sentinel laboratory groups were most reliable at detecting large increases or decreases in the proportion of nonsusceptible invasive isolates; the groups varied in their ability to predict an area’s actual %PNSP; and they were poor at detecting newly emerging fluoroquinolone resistance. As a result, areas considering sentinel surveillance should design systems and interpret data with caution.

Baseline information on isolates processed annually per laboratory and between-laboratory variability in %PNSP can be used to predict how well sentinel systems will perform at estimating this percentage in a given area. Such information can often be collected retrospectively or prospectively from microbiology laboratories. Authorities in areas with high between-laboratory variability or with few isolates per laboratory may want to consider alternatives or complements to sentinel systems.

Reasons for high between-laboratory variability in the proportion of nonsusceptible invasive pneumococcal isolates, such as we observed in Tennessee (Figure), remain unclear. This variability likely reflected differences in the risk for nonsusceptible pneumococcal infections in communities served by different laboratories. Because health insurance policies in the United States often determine the hospitals and laboratories that patients use, these facilities rarely serve populations that are representative of the community as a whole or even the neighborhood where the hospital is located. Characterizing risk factors for nonsusceptible invasive pneumococcal disease in a hospital’s patient population is difficult. Readily obtainable hospital characteristics such as those collected by AHA did not explain the between-laboratory variation we observed. Unfortunately, some known predictors of resistance in health-care settings, such as suburban middle- and upper-class patient populations (*15*,*16*), were not available to link to our surveillance data.

Although most basic hospital characteristics were not a reliable guide to selecting laboratories to be included in sentinel systems, pediatric hospitals were significantly more likely than other hospitals in an area to have a high %PNSP. Because children are a primary reservoir of *S. pneumoniae* and the incidence of invasive pneumococcal disease is elevated in children and the elderly (*1*), states may sometimes choose to include children’s hospitals in sentinel surveillance systems to increase their likelihood of identifying resistance problems. However, to track trends in resistance to drugs such as fluoroquinolones that are not indicated for use in children, children’s hospitals may not be reliable indicators.

For states wishing to increase the reliability of sentinel systems, increasing the overall number of laboratories participating in sentinel systems improved the accuracy of systems, particularly in areas where the %PNSP approaches 50%. However, in areas with high between-laboratory variation in %PNSP, accuracy is difficult to achieve without including most laboratories in the system.

For states or regions with a primary objective of detecting rare, newly emerging resistance profiles, more than one surveillance approach may be necessary. For example, sentinel surveillance combined with universal reporting of fluoroquinolone- or vancomycin-resistant pneumococci will help detect important new strains before they become widespread. Additionally, authorities in such areas may consider collecting the isolates captured by sentinel facilities and conducting susceptibility testing by using a more diverse drug panel than is typically used in most clinical microbiology laboratories.

If used and interpreted appropriately, sentinel laboratory surveillance helps document pneumococcal resistance and improve prevention efforts. Evaluation of alternative surveillance methods such as analysis of hospital antibiograms (*17*) or direct electronic reporting of susceptibility results from hospital laboratories to a central network (M. Soriano-Gabarro, unpub. data) will further contribute to identifying low-cost, feasible methods of documenting trends in pneumococcal resistance.
